# Distillation Time as Tool for Improved Antimalarial Activity and Differential Oil Composition of Cumin Seed Oil

**DOI:** 10.1371/journal.pone.0144120

**Published:** 2015-12-07

**Authors:** Valtcho D. Zheljazkov, Archana Gawde, Charles L. Cantrell, Tess Astatkie, Vicki Schlegel

**Affiliations:** 1 Columbia Basin Agricultural Research Center, Oregon State University, 48037 County 788 Road, Adams, Oregon 97810, United States of America; 2 Natural Products Utilization Research Unit, Agricultural Research Service, United States Department of Agriculture, P.O. Box 8048, University, Mississippi 38677, United States of America; 3 Faculty of Agriculture, Dalhousie University, 50 Pictou Road, P O Box 550, Truro, NS B2N 5E3, Canada; 4 Department of Food Science and Technology, University of Nebraska - Lincoln, 327 Food Technology Complex, Lincoln, Nebraska 68583, United States of America; National Taiwan University, TAIWAN

## Abstract

A steam distillation extraction kinetics experiment was conducted to estimate essential oil yield, composition, antimalarial, and antioxidant capacity of cumin (*Cuminum cyminum* L.) seed (fruits). Furthermore, regression models were developed to predict essential oil yield and composition for a given duration of the steam distillation time (DT). Ten DT durations were tested in this study: 5, 7.5, 15, 30, 60, 120, 240, 360, 480, and 600 min. Oil yields increased with an increase in the DT. Maximum oil yield (content, 2.3 g/100 seed), was achieved at 480 min; longer DT did not increase oil yields. The concentrations of the major oil constituents α-pinene (0.14–0.5% concentration range), β-pinene (3.7–10.3% range), γ-cymene (5–7.3% range), γ-terpinene (1.8–7.2% range), cumin aldehyde (50–66% range), α-terpinen-7-al (3.8–16% range), and β-terpinen-7-al (12–20% range) varied as a function of the DT. The concentrations of α-pinene, β-pinene, γ-cymene, γ-terpinene in the oil increased with the increase of the duration of the DT; α-pinene was highest in the oil obtained at 600 min DT, β-pinene and γ-terpinene reached maximum concentrations in the oil at 360 min DT; γ-cymene reached a maximum in the oil at 60 min DT, cumin aldehyde was high in the oils obtained at 5–60 min DT, and low in the oils obtained at 240–600 min DT, α-terpinen-7-al reached maximum in the oils obtained at 480 or 600 min DT, whereas β-terpinen-7-al reached a maximum concentration in the oil at 60 min DT. The yield of individual oil constituents (calculated from the oil yields and the concentration of a given compound at a particular DT) increased and reached a maximum at 480 or 600 min DT. The antimalarial activity of the cumin seed oil obtained during the 0–5 and at 5–7.5 min DT timeframes was twice higher than the antimalarial activity of the oils obtained at the other DT. This study opens the possibility for distinct marketing and utilization for these improved oils. The antioxidant capacity of the oil was highest in the oil obtained at 30 min DT and lowest in the oil from 360 min DT. The Michaelis-Menton and the Power nonlinear regression models developed in this study can be utilized to predict essential oil yield and composition of cumin seed at any given duration of DT and may also be useful to compare previous reports on cumin oil yield and composition. DT can be utilized to obtain cumin seed oil with improved antimalarial activity, improved antioxidant capacity, and with various compositions.

## Introduction

Cumin (*Cuminum cyminum* L.) is an annual herbaceous plant belonging to family Apiaceae. The plant originates from the Mediterranean region; however, it is presently grown in many regions across the world as a spice and essential oil crop [1, 2]. Due to its unique, pleasant warm and spicy aroma, cumin has been used as spice since ancient times; ancient Greek and Roman cultures utilized it as frequently as pepper and salt are used today. Cumin seed is an essential and main ingredient in many spice mixes or cooking powders, including curry powder, chilli powder, sambar powder, and others [1]. Currently, cumin seed is produced in India, Pakistan, and in most Mediterranean, Eastern European, and in Latin American countries [1, 3]. Cumin seed contains both essential and fatty oil [1, 4]. Cumin essential oil (*Oleum cumini*) is a yellowish fluid with a strong sweet-spicy aroma and is utilized as an aromatic ingredient mainly in the food and liquor industry and to a limited extent in perfumery, cosmetics and pharmaceuticals. Both cumin seed and cumin essential oil have medicinal properties such as antioxidant, cholesterol lowering, and antimicrobial properties [5, 6]. The essential oil of cumin was also reported to have fumigant toxicity against pulse beetle, *Callosobruchus chinensis* [7]. Consequently, cumin seed and cumin essential oil have been utilized in various home-based remedies, especially in the Mediterranean and Asian cultures [1, 3, 5, 8, 9].

The essential oil of cumin is traditionally extracted from cumin seed via steam distillation [2]. However, various authors reported cumin oil composition following different durations of the steam distillation time (DT). There is no agreement in the literature regarding the optimum duration of the DT for maximum oil yield. In addition, it is not clear if the duration of the distillation time would affect cumin essential oil bioactivity or composition. Recently, [10] reported that DT had a significant effect on oil yield and composition of anise seed (*Pimpinella anisum* L.), a plant from the same family. The seeds (fruits) of the two plant species (cumin and anise) has very similar essential oil secretory ducts (vittae). Therefore, our working hypothesis was that the duration of the steam DT would affect essential oil yield, composition, and the bioactivity of cumin seed essential oil.

## Materials and Methods

### Steam distillation and the duration of the steam distillation times (DT)

The cumin seed essential oil was extracted via steam distillation of whole seed using 2-L steam hydrodistillation units (Heartmagic, Rancho Santa Fe, CA) as described previously for anise [10]. The experiment was conducted at the University of Wyoming Sheridan Research and Extension Center in 2013, whereas the essential oil analyses were performed at the USDA-ARS, Natural Product Utilization Research Unit located at the University of Mississippi. The certified bulk cumin seed used in this study originated from India, and was purchased from Starwest Botanicals (Rancho Cordova, CA, U.S.A.).

Ten different distillation times (DT) were performed in this study: 5, 7.5, 15, 30, 60, 120, 240, 360, 480, and 600 min. These DT were based on preliminary studies conducted by the authors, and on literature reports [2]. All DT were performed in 3 replicates, resulting in 30 separate distillations. Each individual sample extracted consisted of 900 g of cumin seed, in order to provide sufficient oil for analyses and for biological evaluations.

At the end of each DT, the cumin oils were collected in glass vials, separated from water, measured on an analytical scale and kept in a freezer until the gas chromatography analyses could be performed. Cumin oil yield (content) was expressed as g of oil per 100 g of cumin seed.

### Gas Chromatography Flame Ionization Detection (GC-FID) Essential Oil Quantitative Analysis

Oil samples were analyzed by GC-FID on a Varian CP-3800 GC equipped with a DB-5 fused silica capillary column (30 m × 0.25 mm, with a film thickness of 0.25 *μ*m) operated using the following conditions: injector temperature, 240°C; column temperature, 60–120 at 3°C/min, then held at 240°C at 20°C/min for 5 min; carrier gas, He; injection volume, 1 *μ*L (split on FID, split ratio 50:1); FID temperature was 300°C.

Commercial standards (-)-β-pinene, (-)-α-pinene, γ-terpinene and cuminaldehyde were obtained from Aldrich (Sigma-Aldrich, Spain) and *p*-cymene was obtained from Fluka (Buchs, Switzerland). With seven concentration points, a least squares regression for quantification was used. Each specific analyte was used to formulate a separate calibration curve using FID response data except for α-terpinene-7-al and β-terpinene-7-al, which were quantitated using the response factor for cumin aldehyde. Linearity was imposed by using response factors (RF) and regression coefficients independently. Response factors (RF) were calculated using the equation RF = DR/C, where DR was the detector response in peak area (PA) and C was the analyte concentration. The chromatograms of each of the essential oil samples from the field experiments were compared to the chromatograms from standards. Target analytes were confirmed by retention time. Confirmed integrated peaks were used to determine percentage of each chemical constituent in the essential oil itself. The RF of the target chemical constituent was used to determine the individual analyte percentage for each sample using the equation PA/RF/C **×** 100 = % analyte in the oil on a wt (analyte)/wt (oil) basis.

### Antioxidant capacity of the oils from different hydrodistillation times (HDT)

The antioxidant capacity of cumin seed oil extracts from 15, 30, 60, 120, 240, 360, 480, and 600 min were measured at the University of Nebraska-Lincoln, Small Molecule Analysis Laboratory, by the oxygen radical absorbance capacity (ORAC_oil_) method [11, 12]. The specifics of the method utilized at the University of Nebraska-Lincoln were detailed previously [10]. Trolox, (6-hydroxy-2,5,7,8-tetramethylchroman-2-carboxylic acid), a polar derivative of Vitamin E, was used as a standard, therefore the results are reported as μmole Trolox g^-1^. The 5 and 7.5 min DT did not yield sufficient oil quantity for antioxidant activity. Each of the oils from the above 8 DT was analyzed in triplicate, hence generating 9 readings for each treatment.

### Antimalarial activity testing of the cumin oils from various DT

The antimalarial activity of the cumin essential oils from the 5, 7.5, 15, 30, 60, 120, 240, 360, 480 and the 600 min DT treatments (all in two replicates) was tested using a method described previously [13] at the National Center for Natural Product Research (NCNPR), The University of Mississippi, University, MS. Additionally, in a secondary late assay, we subjected the pure compounds p-cymene, cuminaldehyde, (-)-beta-pinene, (-)-alpha-pinene, and gamma-terpinene to the same antimalarial assay.

### Antimicrobial activity testing

The antimicrobial testing of the cumin oils was also performed at the NCNPR, University, MS. The primary screening for antimicrobial activity of cumin essential oils obtained from the 5, 7.5, 15, 30, 60, 120, 240, 360, 480 and the 600 min DT treatments (all in two replicates) were tested for antifungal activity against *Candida albicans*, *C*. *glabrata*, *C*. *krusei*, *Aspergillus fumigatus*, *Cryptococcus neoformans* and antibacterial potential against gram +ve bacteria *Staphylococus aureus*, methicillin-resistant *S*. *aureus* and *Mycobacterium intracellulare* and gram–ve bacteria *Escherichia coli* and *Pseudomonas aerogenosa* at a concentration of 50 μg/ml, and % inhibition was calculated following the method published previously [13]. The antifungal activity was tested using ampothericin B, the antibacterial drug control was ciprofloxacin.

### Statistical analyses

The effect of distillation time on oil content (%), and the concentration (%) and yield (mg/100 seed) of α-pinene, β-pinene, γ-cymene, γ-terpinene, cumin aldehyde, α-terpinen-7-al, and β-terpinen-7-al, as well as ORAC (uM Trolox equiv./g) was determined using a one-way analysis of variance. For each response, the validity of model assumptions was verified by examining the residuals as described in literature [14]. The oil content and all yield responses required a transformation to achieve normality assumption, however, the averages shown in the tables are back-transformed to the original scale. Since the effect of distillation time was significant (p-value < 0.05) on all responses, multiple means comparison was completed using Duncan’s multiple range test at the 5% level of significance, and letter groupings were generated. The analysis was completed using the GLM Procedure of SAS [15].

The relationships between distillation time (DT) and oil content (%), and between DT and the yield of β-terpinen-7-al were adequately modelled by the Michaelis-Menten model (Eq 1); and the relationships between DT and the concentrations of α-pinene, β-pinene, γ-terpinene, cumin aldehyde, and α-terpinen-7-al, as well as the relationships between DT and the yields of α-pinene, β-pinene, γ-cymene, γ-terpinene, cumin aldehyde, and α-terpinen-7-al were adequately modelled by the Power model (Eq 2). The relationships between DT and the concentration of γ-cymene and ORAC were very week to describe them by a regression model. The parameters of these nonlinear models (Michaelis-Menten and Power) were estimated iteratively using the NLIN Procedure of SAS [15] and the fitted models met all adequacy requirements [16].

$$Y=\frac{\theta_{1}X}{\theta_{2}+X}+\varepsilon$$ (1)

$$Y=\theta_{1}X^{\theta_{2}}+\varepsilon$$ (2)

Where Y is the dependent (response) variable, X is the independent (DT) variable, and the error term *ε* is assumed to have normal distribution with constant variance. Validity of the normality, constant variance and independence assumptions on the error terms were verified by examining the residuals [16].

## Results

### Effect of distillation time (DT) on oil profile

**Fig 1:**
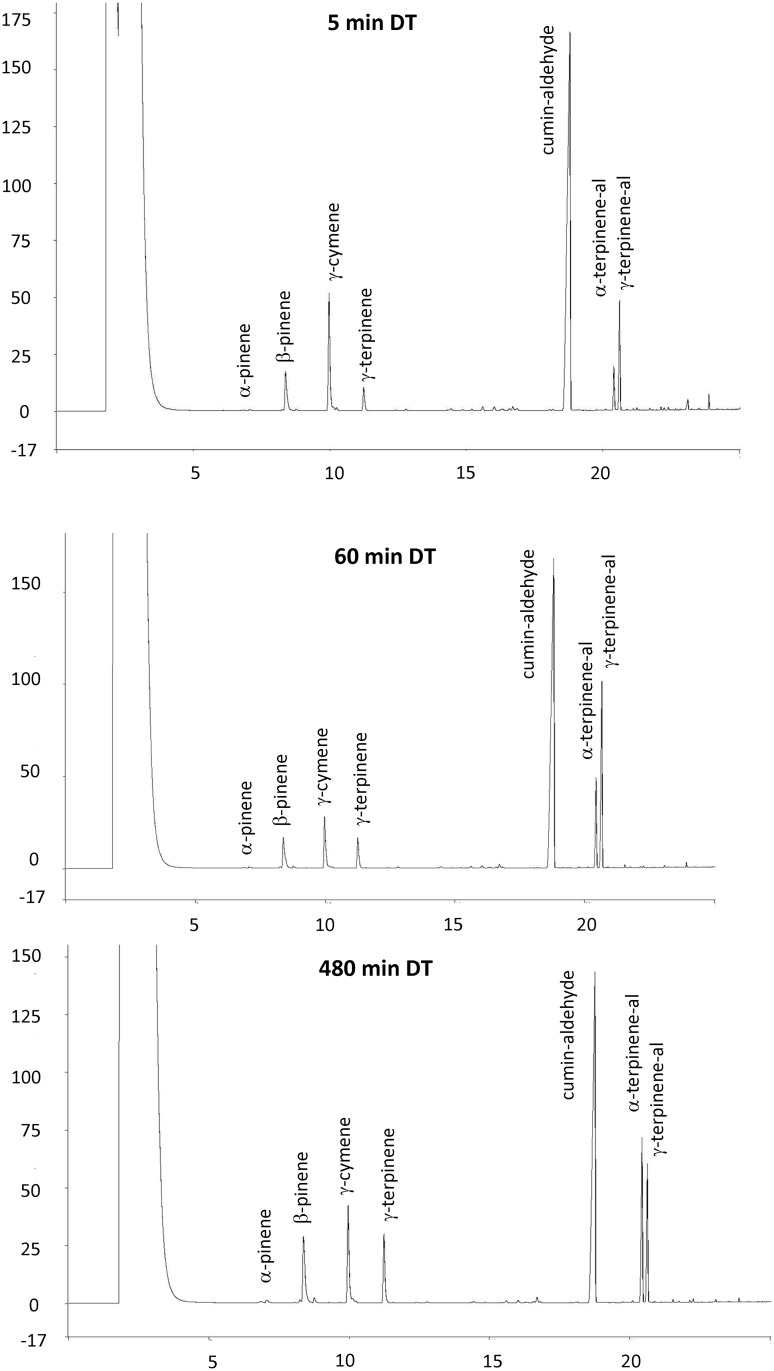
GC-FID Chromatograms of the cumin seed essential oils eluted at 5 min (top) 60 min (middle) and at 480 min DT (bottom).

**Fig 2:**
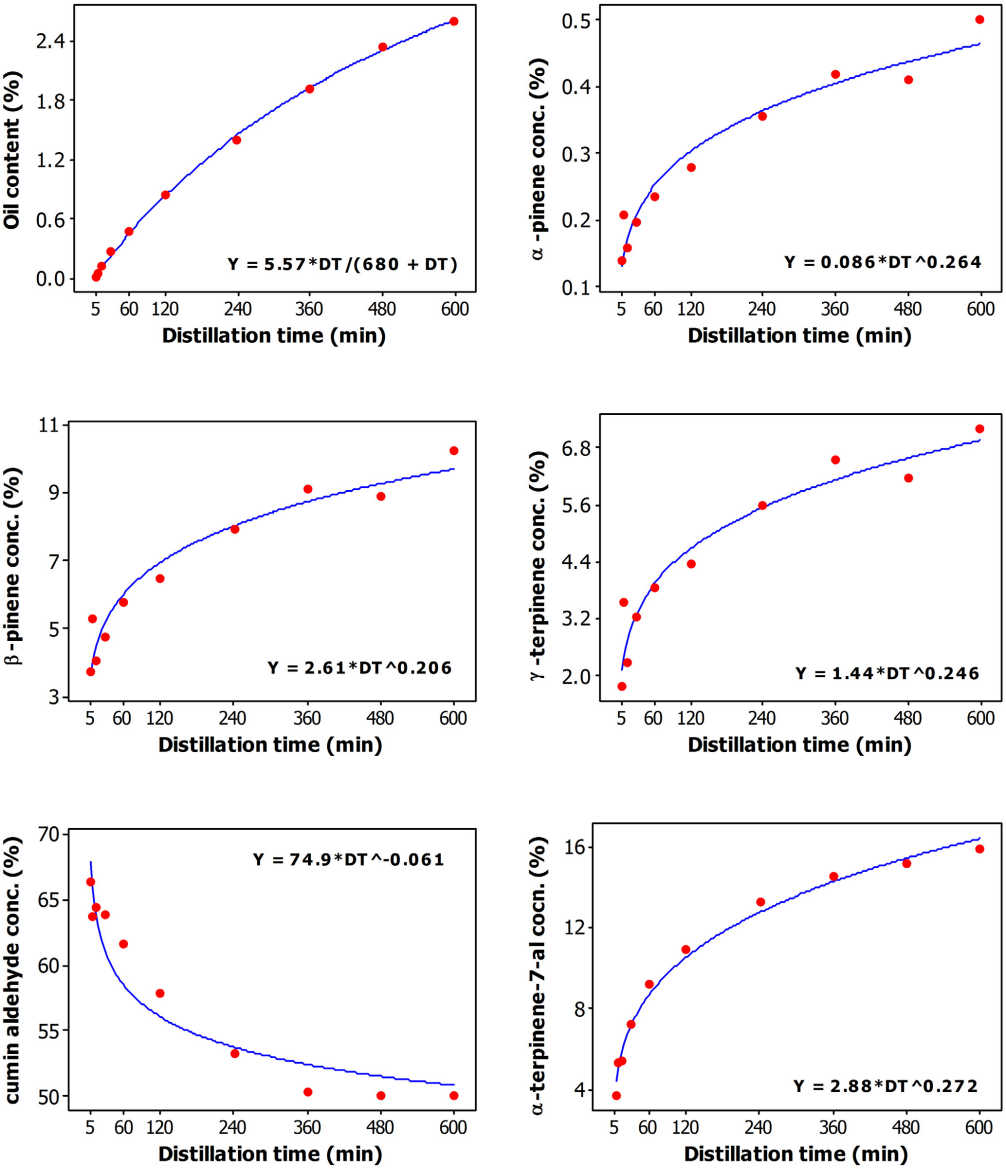
Plot of Distillation time vs oil content (%), and concentration (%) of α-pinene, β-pinene, γ-cymene, γ-terpinene, cumin aldehyde, α-terpinen-7-al, and β-terpinen-7-al along with the fitted (solid line) Michaelis-Menten and Power models.

DT had significant effects on all of the measured responses, and especially on oil profile (Fig 1). Maximum oil yields were obtained at 480 min DT; further increase of DT to 600 min did not result in significant increase of oil yields (Table 1, Fig 2). Generally, the concentrations of α-pinene, β-pinene, γ-cymene, γ-terpinene in the oil increased with the increase in the duration of the DT. The concentration of α-pinene was highest in the oil obtained at 600 min DT (Table 1). Maximum concentrations of β-pinene and γ-terpinene were reached in the oil at 360 min DT; further increase of DT did not significantly change the concentration of these two compounds in the oil (Table 1, Fig 2). The concentration of γ-cymene reached its maximum in the oil at 60 min DT; further increase in the duration of the DT did not bring a corresponding increase of γ-cymene in the oil. The concentration of cumin aldehyde (major cumin oil oil constituent, 50–66% concentration range) was high in the oils obtained at 5–60 min DT, and low in the oils obtained at 240–600 min DT (Table 1, Fig 2). The concentration of α-terpinen-7-al (3.8–15.8% range) was low at shorter DT, increased with the increase of DT and reached maximum in the oils obtained at 480 or 600 min DT. The concentration of β-terpinen-7-al (11.6–20.4% range) was low in 5–15 min DT, reached maximum concentration in the oil at 60 min DT, and decreased after 240 min DT (Table 1, Fig 2).

**Table 1:** Mean oil content (%), and the concentration (%) of α-pinene, β-pinene, γ-cymene, γ-terpinene, cumin aldehyde, α-terpinen-7-al, and β-terpinen-7-al obtained from the 10 distillation times (DT).

DT (min)	Oil content	α-pinene	β-pinene	γ-cymene	γ-terpinene	cumin aldehyde	α-terpinen-7-al	β-terpinen-7-al
5	0.025 h^1^	0.138 e	3.72 f	6.00 abc	1.80 f	66.4 a	3.8 h	11.8 f
7.5	0.055 h	0.207 cde	5.31 def	6.67 ab	3.54 cd	63.7 a	5.3 g	14.9 cde
15	0.120 g	0.159 e	4.07 ef	5.44 bc	2.30 ef	64.5 a	5.4 g	15.6 bcd
30	0.272 f	0.196 de	4.76 def	5.00 c	3.23 de	63.9 a	7.2 f	18.3 abc
60	0.482 e	0.235 cd	5.79 de	5.62 abc	3.86 cd	61.7 ab	9.2 e	20.4 a
120	0.848 d	0.278 c	6.48 cd	5.63 abc	4.34 c	57.8 b	10.9 d	18.6 ab
240	1.406 c	0.356 b	7.95 bc	6.54 ab	5.59 b	53.3 c	13.3 c	16.1 bcd
360	1.920 b	0.419 b	9.14 ab	7.31 a	6.52 a	50.4 c	14.6 b	14.0 def
480	2.333 a	0.410 b	8.89 ab	6.58 ab	6.17 ab	50.0 c	15.1 ab	11.6 f
600	2.603 a	0.500 a	10.28 a	7.33 a	7.18 a	50.0 c	15.8 a	12.3 ef

^1^ Within each column, means sharing the same letter are not significantly different at the 5% level.

### Effect of distillation time (DT) on the yield of individual oil compounds

**Fig 3:**
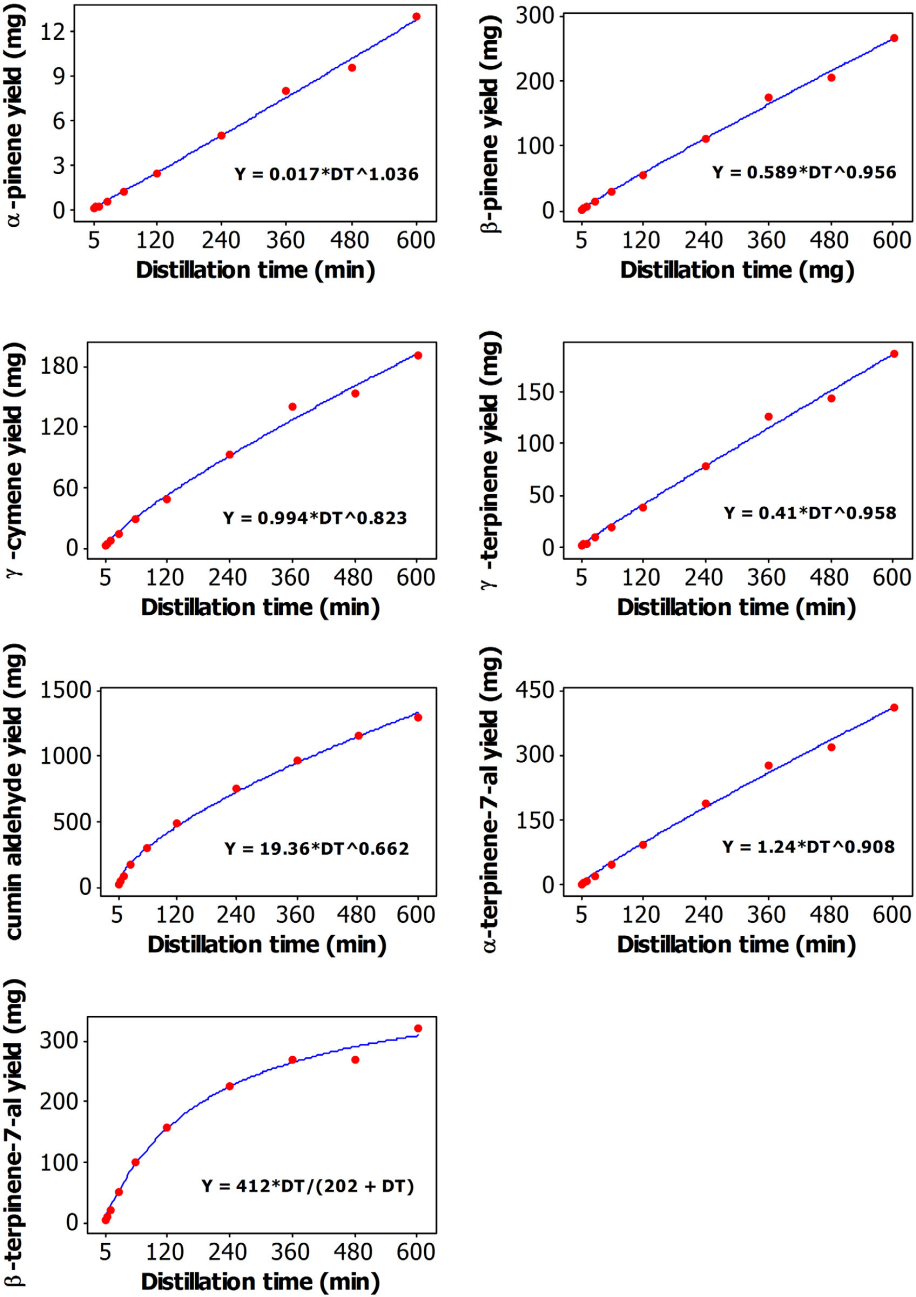
Plot of Distillation time vs yield (mg/100 seed) of α-pinene, β-pinene, γ-cymene, γ-terpinene, cumin aldehyde, α-terpinen-7-al, and β-terpinen-7-al along with the fitted (solid line) Power and Michaelis-Menten models.

The yield of individual oil compounds was calculated from the oil yield and the concentration of given compound in the oil at each DT. Overall, the yield of all essential oil compounds increased with an increase of the duration of the DT and reached maximum at either 600 min DT (α-pinene, β-pinene, γ-cymene, γ-terpinene, and α-terpinen-7-al), or at 480 min DT (cumin aldehyde), or at 360 min DT (β-terpinen-7-al) (Table 2, Fig 3).

**Table 2:** Mean yield (mg/100 seed) of α-pinene, β-pinene, γ-cymene, γ-terpinene, cumin aldehyde, α-terpinen-7-al, and β-terpinen-7-al, antioxidant capacity, ORAC (uM Trolox equiv./g), and antimalarial activity (% inhibition) of the cumin seed oils obtained from the 10 distillation times (DT).

DT (min)	α-pinene	β-pinene	γ-cymene	γ-terpinene	cumin aldehyde	α-terpinen-7-al	β-terpinen-7-al	ORAC	Antimalarial activity
5	0.03 h^1^	0.9 h	1.5 h	0.4 h	17 h	0.9 i	3.0 g	NA	77.0 a
7.5	0.11 g	2.9 g	3.7 g	2.0 g	35 h	2.9 h	8.2 fg	NA	74.5 a
15	0.19 g	4.9 g	6.5 g	2.7 g	77 g	6.5 g	18.8 f	224 bc	31.0 b
30	0.53 f	12.9 f	13.5 f	8.7 f	163 f	19.5 f	49.5 e	338 a	21.0 b
60	1.13 e	27.8 e	27.0 e	18.6 e	298 e	44.5 e	98.6 d	308 ab	28.0 b
120	2.34 d	54.7 d	47.6 d	36.7 d	490 d	92.3 d	158 c	248 abc	34.0 b
240	4.99 c	112 c	91.7 c	78.3 c	749 c	187 c	226 b	269 abc	25.0 b
360	8.02 b	175 b	140 b	125 b	966 b	279 b	269 ab	202 c	19.0 b
480	9.52 b	206 b	153 b	143 b	1162 a	321 b	269 ab	304 ab	36.0 b
600	13.01 a	267 a	191 a	187 a	1301 a	412 a	320 a	249 abc	37.0 b

^1^ Within each column, means sharing the same letter are not significantly different at the 5% level.

### Effect of distillation time (DT) on antioxidant capacity and on antimicrobial activities

The antioxidant activity of the oils from this study varied from 202 uM Trolox equiv./g (in the oil from 360 min DT) to 338 uM Trolox equiv./g (in the oil from the 30 min DT) (Table 2).

Cumin oils obtained during the 0–5 and 5–7.5 min DT timeframes showed high antimalarial activity; 77 and 75% respectively, suppression of *Plasmodium falciparum* (Table 2). The antimalarial activity of these oils was significantly higher (twice as much) compared to the antimalarial activity of the oils from the other DT (Table 2). However, in a later assay, the pure commercially available compounds did not show antimalarial activity against *P*. *falciparum* up to a concentration of 12 μg/ml (which was 2.5 times higher than our routine testing concentration for pure compounds).

The antimicrobial activity of the cumin oils from various DT was not significant against most of the organisms with the exception of *Cryptococcus neformans*. The cumin oils obtained at 0–5 and at 0–7.5 min showed above 50% activity against *Cryptococcus neformans* in the primary screening. The activity of the cumin oils obtained from the later DT showed between 20 and 50% activity against *Cryptococcus neformans*.

Nonlinear regression analysis of the data revealed that the relationship between DT and oil content can be described by the Michaelis-Menton model (Fig 2). The relationship between DT and β-terpinene-7-al yield was also very well described by the Michaelis-Menton model (Fig 3). The parameters shown in the last plot of Fig 3 suggest that the maximum β-terpinene-7-al yield that can be achieved is 412 mg, and the distillation time required to achieve half of this maximum yield is 202 min. As shown in Figs 2 and 3, the other relationships were described by the Power model.

## Discussion

This is the first report on the effect of DT on cumin whole seed oil yield, oil composition, on cumin oil antimalarial activity, and on cumin oil antioxidant activity. DT has been recently shown to significantly change essential oil yield and composition of oil from anise seed [10], from biomass of crops from other families such as peppermint (*Mentha ×piperita* L.), lemongrass (*Cymbopogon flexuosus* Steud.), palmarosa (*Cymbopogon martinii* Roxb.) [17] and from lavender [18], among other crops.

The concentration of the seven major oil constituents (α-pinene, β-pinene, γ-cymene, γ-terpinene, cumin aldehyde, α-terpinen-7-al, and β-terpinen-7-al) varied significantly as a function of the DT, instead of their boiling points. Although it may seem logical that concentrations of constituents would vary as a function of their boiling points, there are many additional factors that have a greater influence on final concentration and yield. This is not a true distillation of the oil but rather steam at 100 C is passed through the plant material volatilizing these constituents. Furthermore, the constituents in the tissue may be available for extraction by the steam to varying degrees creating these differences in concentration unrelated to boiling point. This is a distillation process that mimic the process employed by the commercial oil extraction facilities.

In this study, the oil from 600 min DT had the highest concentration of α-pinene compared to the oils from the other DT. The oils obtained at 360 min were characterized with the highest concentrations of β-pinene and γ-terpinene. The oil at 60 min had the highest concentration of γ-cymene compared to the other oils. The concentration of cumin aldehyde (the main oil constituent) was high in the oils obtained at 5–60 min DT, and low in the oils obtained at 240–600 min DT. Furthermore, α-terpinen-7-al reached maximum in the oils obtained at 480 or 600 min DT, whereas β-terpinen-7-al reached maximum concentration in the oil at 60 min DT. Generally, the yields of all oil constituents increased with the increase in DT and reached their respective maximums at 480 or 600 min DT, which was due to increase in essential oil yield and the increased concentration of some individual compounds in the oil.

The cumin seed oils in the 5 and in the 7.5 min DT had twice the antimalarial activity of the oils from the other DT. This opens the possibility for distinct marketing and utilization for these improved oils. Currently, the plant that is used for malaria treatment is *Artemisia annua* L. (Asteraceae), because it contains the active ingredient artemisinin, which has proven antimalarial activity. *Artemisia annua* is currently the only commercial source for production of artemisinin-based combination therapies (ACT) [19, 20]. It is estimated that around 2 million doses of artemisinin from *A*. *annua* are needed on yearly basis to be used as the first line of defense against multi-drug-resistant *Plasmodium falciparum* malaria, as recommended by the World Health Organization [20]. Cumin seed oil with high antimalarial activity may be a welcoming new antimalarial agent. Indeed, there is a pending patent on the use of cumin seed powder and cumin oil as capsules for malaria treatment [21]. However, in this study we describe a method that can produce cumin oil with twice as much inhibition power than the common cumin oil. The cumin oil with improved antimalarial activity may render greater acceptance and utilization as it comes from a spice, edible plant.

In a follow up later assay, the pure commercially available compounds (p-cymene, cuminaldehyde, (-)-β-pinene, (-)-α-pinene, and γ-terpinene) did not show antimalarial activity against *P*. *falciparum*. Therefore, minor constituents are likely responsible for the antimalarial activity. A full bioassay-directed fractionation will be performed but it is the subject of a more intensive study and not part of this study.

DT in this study affected the antioxidant activity of cumin seed oil, with the highest antioxidant capacity of the oil obtained at 30 min DT and the lowest in the oil from 360 min DT. Recent studies found that DT affected antioxidant activity of oils of other crops such as wormwood, *Artemisia annua* [22], fennel, *Foeniculum vulgare* Mill. biomass [23], Rocky mountain juniper, *Juniperus scopulorum* Sarg. [24]. Hence, when antioxidant activity of a certain oil is reported, DT must also be reported.

The regression models developed in this study can be utilized to predict essential oil yield and composition of cumin seed at any given duration of DT. The regression models could also be useful to compare data from reports on cumin oil yield and composition. This study demonstrated DT can be utilized to obtain cumin seed oil with various compositions and bioactivity from the same batch of seed.
